# Retinal prosthesis edge detection (RPED) algorithm: Low-power and improved visual acuity strategy for artificial retinal implants

**DOI:** 10.1371/journal.pone.0305132

**Published:** 2024-06-18

**Authors:** Yeonji Oh, Jonggi Hong, Jungsuk Kim

**Affiliations:** 1 Department of Medical Science, Korea University, Seoul, South Korea; 2 Department of Health Sciences & Technology, Gachon Advanced Institute for Health Sciences & Technology, Gachon University, Incheon, South Korea; 3 Department of Biomedical Engineering, Gachon University, Sungnam, South Korea; 4 Cellico Research and Development Laboratory, Sungnam, South Korea; Islamia University of Bahawalpur: The Islamia University of Bahawalpur Pakistan, PAKISTAN

## Abstract

This paper proposes a retinal prosthesis edge detection (RPED) algorithm that can achieve high visual acuity and low power. Retinal prostheses have been used to stimulate retinal tissue by injecting charge via an electrode array, thereby artificially restoring the vision of visually impaired patients. The retinal prosthetic chip, which generates biphasic current pulses, should be located in the foveal area measuring 5 mm × 5 mm. When a high-density stimulation pixel array is realized in a limited area, the distance between the stimulation pixels narrows, resulting in current dispersion and high-power dissipation related to heat generation. Various edge detection methods have been proposed over the past decade to reduce these deleterious effects and achieve high-resolution pixels. However, conventional methods have the disadvantages of high-power consumption and long data processing times because many pixels are activated to detect edges. In this study, we propose a novel RPED algorithm that has a higher visual acuity and less power consumption despite using fewer active pixels than existing techniques. To verify the performance of the devised RPED algorithm, the peak signal-to-noise ratio and structural similarity index map, which evaluates the quantitative numerical value of the image are employed and compared with the Sobel, Canny, and past edge detection algorithms in MATLAB. Finally, we demonstrate the effectiveness of the proposed RPED algorithm using a 1600-pixel retinal stimulation chip fabricated using a 0.35 μm complementary metal-oxide-semiconductor process.

## Introduction

**Fig 1:**
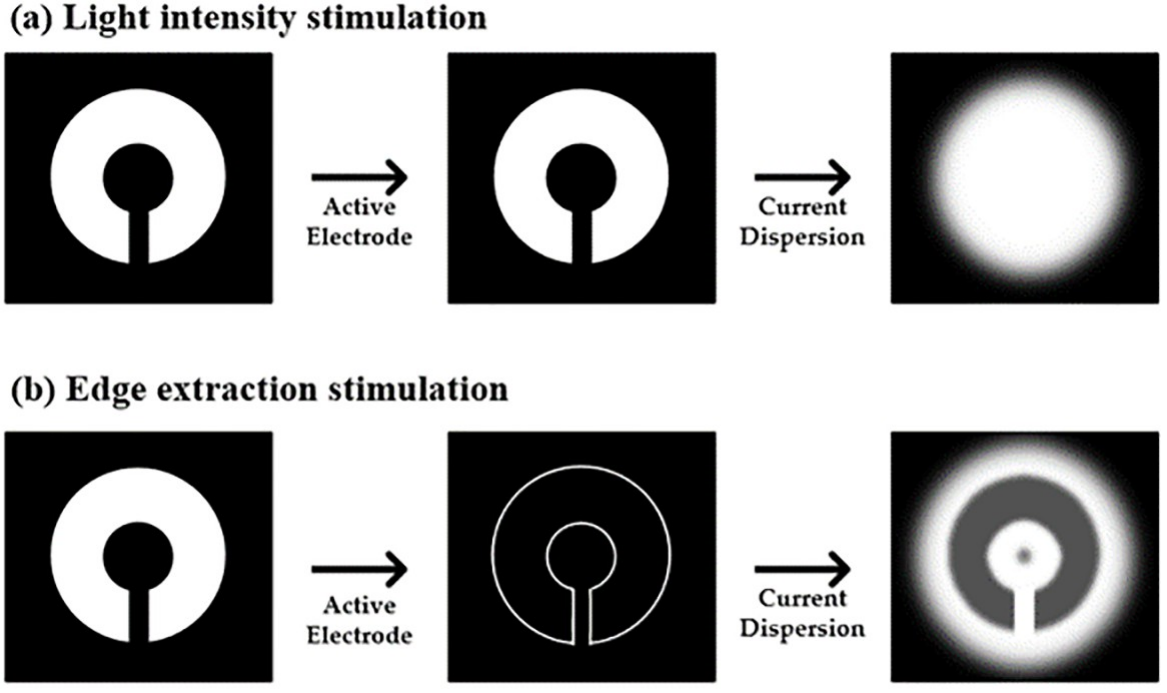
Stimulation methods of (a) light intensity (b) edge extraction.

An artificial retina, also known as a retinal prosthesis, is a device used to restore vision in individuals who have lost their eyesight because of specific degenerative eye conditions, such as retinitis pigmentosa (RP) and age-related macular degeneration (AMD) [1, 2]. These diseases damage the retinal photoreceptor layer, which detects light and transmits visual signals to the brain, resulting in vision loss. However, retinal neurons in the inner nuclear and ganglion cell layers in the macula survive at a high rate [3]. A retinal prosthesis replaces photoreceptors with an electrode array that delivers electrical stimulation [4, 5]. It stimulates the remaining healthy retinal cells, transmitting visual information to the brain and restoring vision [6, 7]. Retinal prostheses are categorized into two main types: epiretinal and subretinal. An epiretinal prosthesis is implanted in the innermost layer of the retina, directly stimulating the ganglion cells [8, 9]. A subretinal prosthesis stimulates bipolar cells by inserting microelectrode arrays into the photoreceptor cell layer [10, 11]. Moreover, it has been reported that the subretinal prosthesis can be manufactured as a high-resolution pixel chip within a limited retinal fovea size (5 mm × 5 mm) owing to simple analog circuit implementation. A subretinal prosthesis requires high-resolution pixels within a limited retinal fovea size to recognize a person or object [12]. In this case, the proximity of the pixels leads to interference between neighboring pixels during stimulation, resulting in current dispersion and the creation of a blurred image, as illustrated in Fig 1(a). Since the pixel count in a subretinal stimulator aligns with the number of electrodes [13], this equivalence can also be interpreted as interference from the electrodes. Fig 1 depicts the results consisting of 2025 electrodes with a spacing of 10um, and the current dispersion may vary depending on the performance of the stimulator. Furthermore, heat is generated when a current is applied to the tissue owing to tissue resistance, which can damage retinal cells and surrounding tissues [14, 15]. In other words, as the number of current emitting pixels increases, the probability of cell damage increases because of the high thermal effect.

To overcome these problems as illustrated in Fig 1(a), edge detection methods have been proposed to selectively stimulate only the edge regions, as depicted in Fig 1(b). Edge detection stimulation extracts the intensity value of a pixel and compares it with the mean value of neighboring pixels. By comparison, if the pixel has a difference above a threshold value, the pixel was determined to be an edge. Utilizing edge detection stimulation in retinal prostheses offers several advantages: (1) The power consumption of the retinal prosthesis system can be reduced by selectively activating electrodes that correspond to edges. Generally, implantable retinal prosthesis power is supplied via wireless power transmission. When applying wireless power transmission, it is crucial to consume less power because of safety issues related to the heating effect and radiation [16]. (2) Crosstalk between pixels is geometry-dependent and characteristic of monopolar configurations [17]. This selective electrode activation method reduces the electric crosstalk between electrodes, which induces current dispersion [18], by reducing the number of activated electrodes. (3) As edge detection closely resembles natural retinal visual signal processing [19], visual acuity can be improved using an edge detection method that limits stimulation of electrodes along the edges [20–22].

Applying the edge detection method to retinal prostheses requires tailored image processing that is suitable for small retinal stimulation chips, unlike conventional computer-based image processing, which typically operates for sufficient durations. First, an image processor with an algorithm suitable for low-resolution, rather than high-resolution, should be integrated into the chip. In contrast to computer-based edge-detection methods, which typically handle high-resolution images with millions of pixels, retinal prostheses are limited to low-resolution images with thousands of pixels because of the spatial restrictions of the eye. Second, real-time processing capabilities are essential for patients’ daily lives. It has been known that retinal cells are stimulated by a temporal resolution corresponding to 40–100ms (25–10 Hz) [23, 24]. Finally, a minimum number of electrodes should be activated for visual recognition. Typically, power to the retinal prosthesis is supplied via wireless power transmission. However, it is difficult to supply sufficient power to wireless power transmissions currently in use owing to problems such as eddy effects, proximity issues, misalignment, and large separations [25–27]. Therefore, power consumption is minimized to ensure reliable operation, even under less-than-optimal power transmission conditions.

To implement edge detection, the internal image processor of the chip compares the intensity of the pixels with the surrounding pixels to discern the edge. In a retinal prosthesis, conventional edge detection compares the center pixel with its four neighboring pixels, and the five pixels are simultaneously activated [22]. The image processing speed is slow because when more pixels are activated, the power consumption and amount of data to be processed are higher than those of conventional edge detection. To overcome these issues, this paper introduces an effective ‘retinal prosthesis edge detection (RPED)’ algorithm optimized for retinal prosthesis by activating only two neighboring pixels, thereby reducing power consumption and increasing data processing speed. We mathematically model and simulate the proposed algorithm and compare and validate it with conventional edge detection algorithms, such as Canny and Sobel, using various evaluation metrics, including the mean squared error (MSE), peak signal-to-noise ratio (PSNR), and structural similarity index map (SSIM). Additionally, we demonstrate the RPED algorithm on an actual 1,600 pixel artificial subretinal chip [28].

## Method

**Fig 2:**
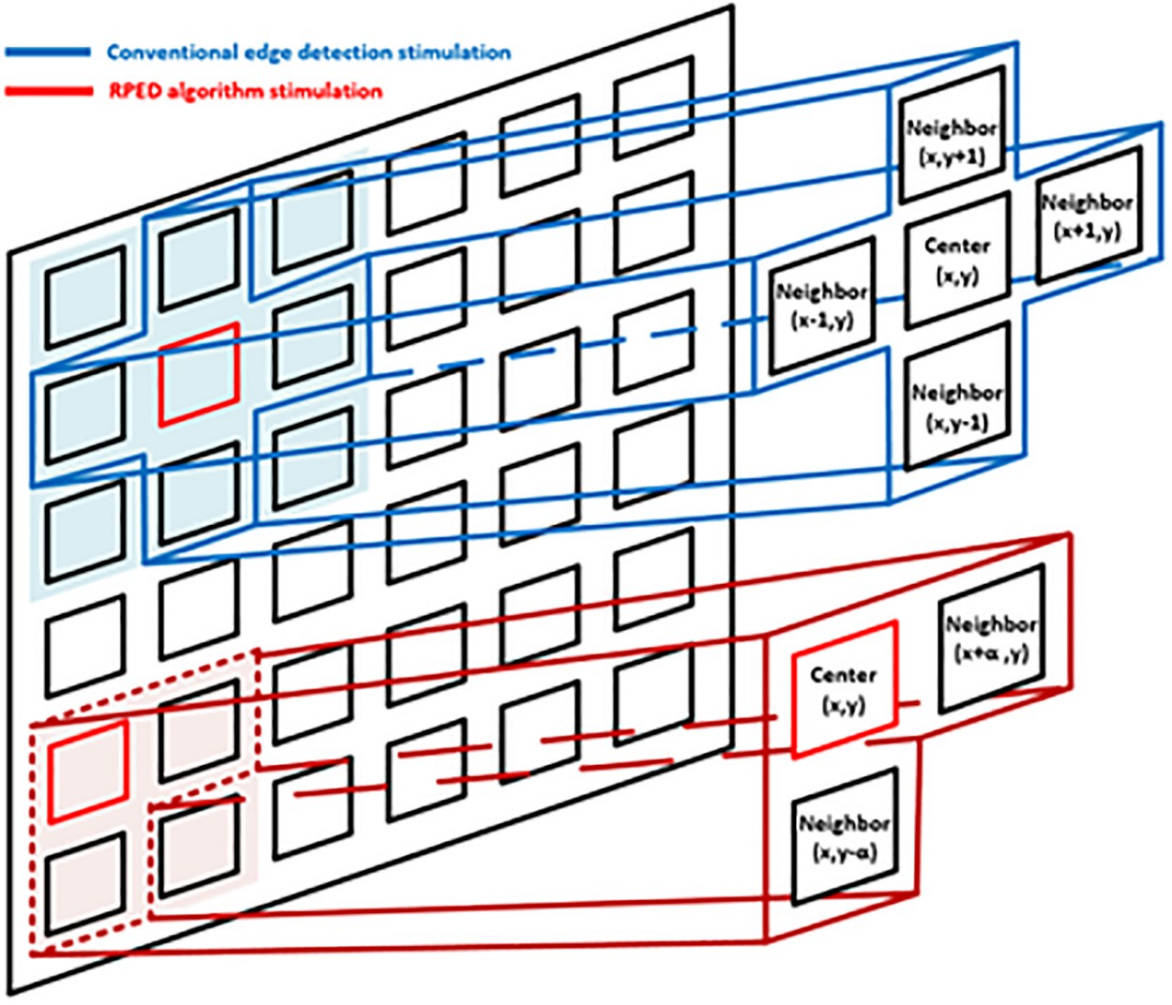
Comparison of the principle between conventional edge detection stimulation (blue) and RPED algorithm stimulation (red).

Fig 2 compares the pixels of the edge detection stimulation between the conventional retinal prosthesis (blue line) and the RPED algorithm (red line). In conventional edge detection stimulation methods, edge detection is performed by comparing the information from the central pixel with that from its four surrounding pixels. If the center pixel has a difference above a threshold value compared with the neighboring pixels, the center pixel is judged to be an edge and is activated. This process has the disadvantage of increasing power consumption and reducing data processing speed while the surrounding pixel values are collected. Therefore, this study proposes an RPED algorithm that reduces power consumption and increases data processing speed by detecting edges using only two neighboring pixels.

**Fig 3:**
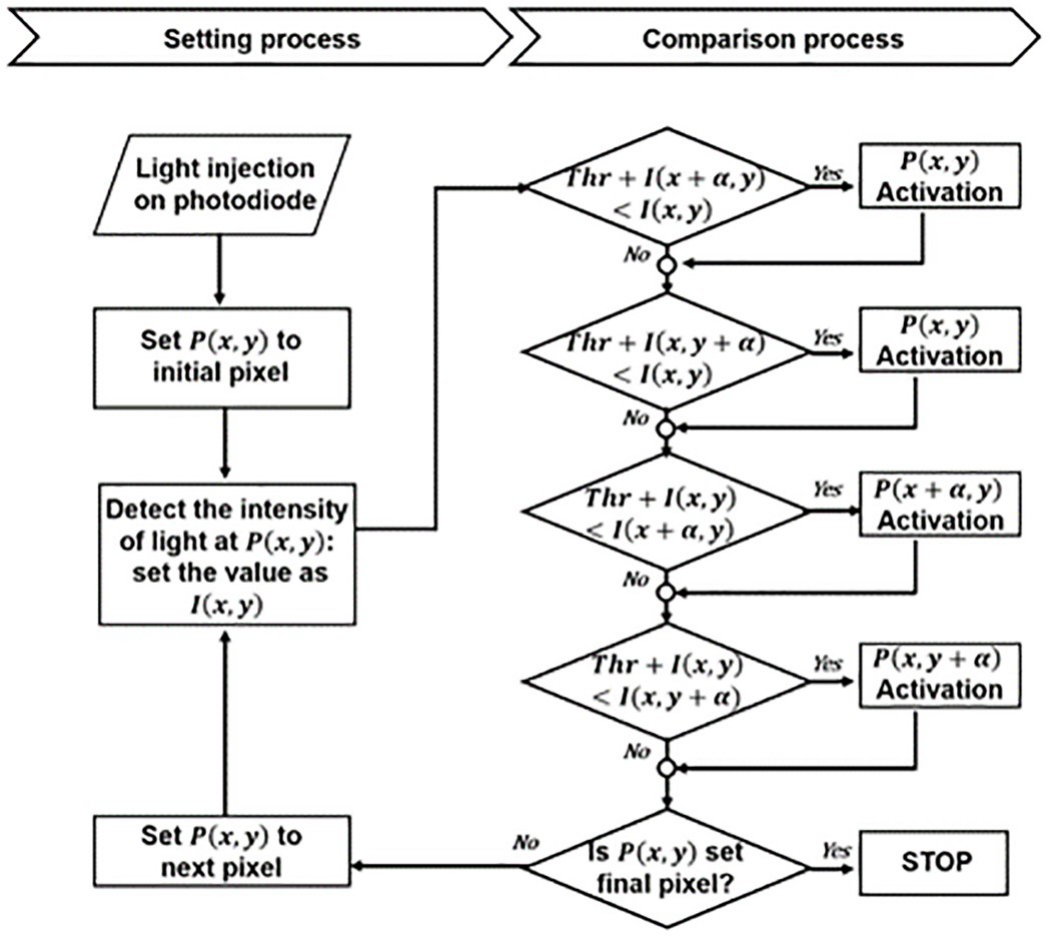
Flow chart of the proposed RPED algorithm.

Fig 3 illustrates a flowchart of the proposed RPED algorithm, and *P* presents the pixel. When light is injected into a photodiode-based retinal stimulator chip, the algorithm begins with the initial pixels and then proceeds sequentially to the next activated pixels. First, as shown in Eq 1, a comparison between the center pixel and its two neighboring pixels was initiated.


$$I\left(x,y\right)>Thr+I\left(x+\alpha ,y\right)\;||\;I\left(x,y\right)<Thr+I(x,y+\alpha )$$ (1)

In Eq 1, *I*(*x*,*y*) represents the amount of dark current flowing in the center-pixel photodiode. *Thr* is the threshold that denotes a certain level of luminance difference. Additionally, *α* signifies the distance between the center pixel and neighbor pixels, which in Fig 2 can be defined as *α* = 1.

If the center pixel has a brighter light than the neighboring pixel, resulting in a luminance difference exceeding the user-defined threshold, the center pixel is identified as an edge and is activated. Conversely, if the center pixel has a minimal luminance difference and was considered insufficient for recognition as an edge, it was deactivated. However, considering the possibility of edges with varying luminance differences, where neighboring pixels may be brighter than the center pixel, both *x*-axis and *y*-axis comparisons were conducted, as described in Eqs 2 and 3.


$$Thr+I\left(x,y\right)<I\left(x+a,y\right)$$ (2)

$$Thr+I\left(x,y\right)<I\left(x,\;y+a\right)$$ (3)

In this process, neighboring pixels identified as edges are activated, whereas non-edge pixels are deactivated. Following the edge determination of the initial pixel, a comparison process for the next pixel was carried out. During this process, once a pixel has been activated, it retains its activation status even if it is not recognized as an edge in subsequent comparisons. This sequence continues until all pixels have undergone an edge assessment. As the algorithm determines the activation/deactivation of a pixel, a charge proportional to the light intensity is applied to the cell, enabling the creation of gray-level images that closely resemble the original input. Retinal prostheses typically contain over 2k high-resolution pixels, resulting in the presence of multiple pixel clusters [29]. Activating a large number of pixels simultaneously may increase power consumption through wireless power transmission used in implantable devices, potentially leading to heat generation as explained in the introduction. For these reasons, several initial pixels are designated for concurrent edge detection within multiple clusters.

The performance of edge detection algorithms is typically evaluated using metrics, such as the PSNR and SSIM, based on the extracted edge image. The PSNR assesses the quality loss in an image and is calculated using Eq 4, where R represents the maximal variation in the input image data, typically having a value of 255 for 8-bit unsigned integer data types [30].


$$PSNR=10\;{\text{log}}_{10}\left(\;\frac{R^{2}}{MSE}\;\right)$$ (4)

The MSE in Eq 5 represents the average of the squared differences between the predicted values of the algorithm and the actual image data. I1 is the original image, I2 is the edge-detected image, and m and n are the height and width of the image, respectively [30]. A lower MSE indicates a better algorithm performance, resulting in a higher PSNR. In other words, the PSNR increases as the error rate decreases.


$$MSE=\frac{\sum_{M,N}{\left[I_{1}(m,n)\;-\;I_{2}(m,n)\right]}^{2}}{M,N}$$ (5)

The SSIM evaluates human visual quality differences rather than numerical errors, as in Eq 6, and represents the quality differences that humans feel visually, unlike PSNR [31].


$$SSIM\left(x,\;y\right)=\frac{\left(2\mu_{x}\mu_{y}+c_{1}\right)\left(2\sigma_{xy}+c_{2}\right)}{\left(\mu_{x}^{2}+\mu_{y}^{2}+c_{1}\right)\left(\sigma_{x}^{2}+\sigma_{y}^{2}+c_{2}\right)}$$ (6)

Here, *μ*_*x*_ denotes the pixel sample mean of *x*, *μ*_*y*_ denotes the pixel sample mean of $y,\;\sigma_{x}^{2}$ indicates the variance of $x,\;\sigma_{y}^{2}$ denotes the variance of *y*, and *σ*_*xy*_ denotes the covariance of *x* and *y*. Additionally, *c*_1_ and *c*_2_ are defined as, *c*_1_ = (*k*_1_*L*)^2^, *c*_2_ = (*k*_2_*L*)^2^ with *k*_1_ = 0.01, *k*_2_ = 0.03. *L* signifies the dynamic range of pixel values, typically defined as 2^#*bits per pixel*^−1. Based on these evaluation metrics, algorithms were implemented and compared through MATLAB to evaluate the performance of the proposed RPED algorithm and to compare it with conventional edge detection stimulation methods and other edge detection algorithms such as Canny and Sobel.

## Result

### 3.1 Simulation results with MATLAB

For experiments under the same conditions as the retinal prosthesis stimulator with 2,000 or more pixels, the original image was converted into a grayscale image with a resolution of 45 × 45 pixels. Lenna images, which are commonly used in image processing algorithms, were used.

#### 3.1.1 Retinal prosthesis edge detection (RPED) algorithms

To optimize the RPED algorithm for the Lenna image, the variables Threshold and *α* should be defined. To simplify the determination of the threshold value, we divided the pixel values of an image, ranging from 0 to 255, into 15 discrete steps. For the Lenna image, which had values ranging from 24 to 229, we divided these values into 15 steps, resulting in intervals of 14. To simplify threshold naming, we assign ‘*Thr*’ values proportionally based on pixel value differences, such as *Thr* = 1 for a difference of 14 in pixel values and *Thr* = 2 for a difference of 28. Table 1 lists the results of the edge detection image based on the change in the value of *Thr*. We optimized the Threshold to *Thr* = 4 for the Lenna image, ensuring that the PSNR, which is inversely proportional to power consumption, is high and prevents the blur effect caused by current dispersion. *Thr* can vary depending on the patient’s clarity through presetting of a transplant operation by using external digital code. The fixed *Thr* value remains constant during the operation of the stimulator. However, in this experiment, *Thr* is tailored to the image.

**Table 1:** Results of edge detection image based on the *Thr* value change.

Table1-1	Table1-2	Table1-3	Table1-4	Table1-5	Table1-6	Table1-7	Table1-8
**Original (*Thr* = 0)**	*Thr* = 1	*Thr* = 2	*Thr* = 3	*Thr* = 4	*Thr* = 5	*Thr* = 6	*Thr* = 7
**PSNR**	14.1925	10.8681	9.5410	8.8613	8.3422	7.8619	7.4740
**SSIM**	0.6368	0.5003	0.4095	0.3441	0.2813	0.2254	0.1704
**Activated pixel N**	1459	984	719	575	442	331	243
**Activated pixel ratio**	72.05%	48.59%	35.51%	28.4%	21.83%	16.35%	12%

**Fig 4:**
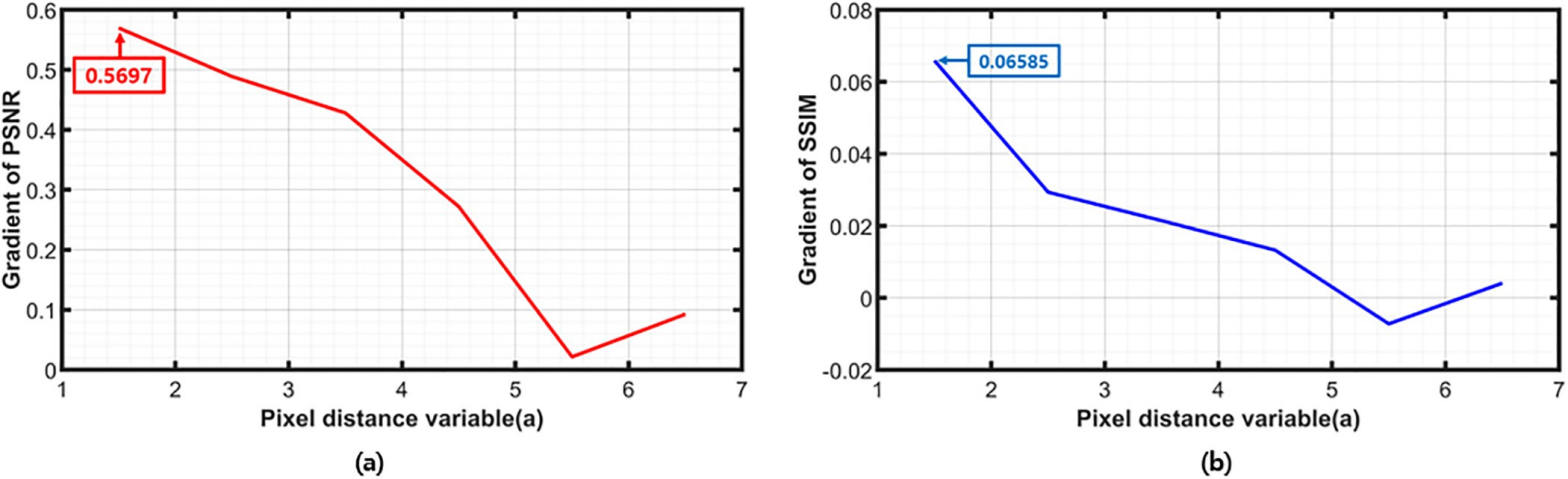
Graph of (a) pixel distance variable vs gradient of PSNR (b) pixel distance variable vs gradient of SSIM.

Table 2 lists the results based on the change of α, which is the distance variable between pixels when the threshold is fixed at 4. The algorithm first determines the Thr and then optimizes the *α* value, and this process occurs during presetting. The PSNR value increases gradually but is inversely proportional to the power. Therefore, it is optimized at *α* = 2, where the most significant increase rate of PSNR exists. Fig 4(a) presents a graph generated based on the PSNR values defined in Table 2, illustrating the increase rate of PSNR as *α* increases. It is evident that the most substantial increase rate occurs at *α* = 2, reaching 0.5697. Fig 4(b) displays the increase rate of SSIM, with *α* = 2 exhibiting the highest increase rate at 0.06585. These results confirm that the optimal choice is *α* = 2, representing the distance between pixels.

**Table 2:** Results of edge detection image based on the *α* value change.

Table2-1	Table2-2	Table2-3	Table2-4	Table2-5	Table2-6	Table2-7	Table2-8
**Original**	*α* = 1	*α* = 2	*α* = 3	*α* = 4	*α* = 5	*α* = 6	*α* = 7
**PSNR**	7.7219	8.8613	9.8389	10.6950	11.2394	11.2826	11.4680
**SSIM**	0.2124	0.3441	0.4028	0.4457	0.4722	0.4578	0.4660
**Activated pixel N**	364	575	711	834	901	910	944
**Activated pixel ratio**	17.83%	28.4%	35.11%	41.19%	44.49%	44.94%	46.62%

#### 3.1.2 Comparison with other algorithms

The results of comparing the optimized RPED algorithm with conventional edge detection stimulation of retinal prostheses are summarized in Table 3. The PSNR and SSIM show that the proposed algorithm has a higher value than the conventional edge detection method when both images have a resolution of 45 × 45 pixels, as previously assumed. The power consumption is calculated mathematically by assuming the environment of the retinal prosthesis stimulator chip as 5 V of supply voltage and 100 μA of stimulation current for retina tissue. In the RPED algorithm, the activated pixel power of the final image is higher than that of the conventional edge detection stimulation because the number of activated pixels is greater. However, because the algorithm was applied to a retinal prosthesis, the processing power consumption during the edge detection of pixels should be considered. In this 45 × 45-pixel case, because edge detection starts from several initial pixels in multiple clusters, as mentioned in the Methods section, suppose that the initial pixels are activated in 25 clusters. The 25 clusters were created by dividing the 45 × 45 blocks into 9 × 9 blocks. The numbers of pixels required to determine the edge are 3 and 5, respectively, which are the sum of the center and neighboring pixels, respectively. In the proposed algorithm, three pixels were activated in each of the 25 blocks, resulting in a processing power consumption of 37.5 mW as shown in Table 3. By contrast, five pixels were activated in each block of the conventional edge detection stimulation methods, leading to a processing power consumption of 62.5 mW. Although the power consumption results may vary depending on the specifications of the retinal prosthesis, the number of activated pixels required to determine the edge of a single center pixel remains the same. Therefore, the proposed RPED algorithm consistently exhibits lower processing power consumption and is safer from cell damage due to heat than the conventional edge detection stimulation. Additionally, it is assumed that the time required to detect the edge of a single pixel for data processing speed calculation is 2 μs. The proposed RPED algorithm only requires comparison with two neighboring pixels, thus necessitating 4 μs per pixel. Therefore, for all 2025 pixels, the total time required amounts to 8.1ms. Using the same approach, for the conventional edge-detection stimulation method, which requires comparison with four neighboring pixels, twice the time of the former is needed, totaling 16.2 ms. The halved comparison time enables more feasible real-time image processing. The calculation results in the environment where the values of *α* and Thr were set equally for fair comparison between the two are listed in Table 3.

**Table 3:** The results of comparing the RPED algorithm with conventional edge detection stimulation.

	Retinal prosthesis edge detection algorithm	Conventional edge-detection stimulation method
**Number of neighbor pixels**	2	4
**Image result**	Table3-1	Table3-2
**PSNR**	8.8613	7.5862
**SSIM**	0.3441	0.2034
**Activated pixel N**	575	309
**Activated pixel ratio**	28.4%	15.26%
**Activated pixel power**	287.5 mW	154.5 mW
**Processing power**	37.5 mW	62.5 mW
**Data processing speed**	8.1ms	16.2ms

Table 4 compares the RPED algorithm with two well-known image processing algorithms, Sobel [32] and Canny [33], to assess its suitability for retinal prosthesis systems. The comparison was conducted under the resolution conditions of an artificial retina with dimensions of 45 × 45 pixels and under the original image resolution of 512 × 512 pixels. Sobel edge detection calculates the image gradient using a 3 × 3 convolution filter to detect edges [34]. Canny edge detection involves several stages including smoothing using a Gaussian filter, gradient calculation, non-maximum suppression (NMS), and hysteresis edge tracking to achieve precise edge detection and noise reduction [35]. Both methods yielded excellent edge detection results in high-resolution images. However, in the case of low-resolution conditions such as retinal prostheses, the resolution of the edge decreases, making edge discrimination challenging. Additionally, both methods share the drawback of weak antinoise capability, which may classify noise as edges, thus emphasizing noise [36]. Examining the results in Table 4, it is evident that, for high-resolution images (512 × 512 pixels), similar PSNR values were observed among the algorithms. However, for low-resolution images (45 × 45 pixels), the RPED algorithm achieves a higher PSNR. Furthermore, the RPED algorithm exhibits superior SSIM values. In summary, these results indicate that the proposed RPED algorithm is well-suited for implantable retinal prosthesis systems that require low-resolution and ultra-low power operation.

**Table 4:** Comparison with RPED algorithm vs Sobel and Canny algorithm.

Algorithm	Sobel	Canny	RPED
**45 × 45 Resolution**			
**Image result**	Table4-1	Table4-2	Table4-3
**PSNR**	6.5718	7.0599	8.8613
**SSIM**	0.0188	0.0436	0.3441
**512 × 512 Resolution**			
**Image result**	Table4-4	Table4-5	Table4-6
**PSNR**	5.9168	6.1159	6.2742
**SSIM**	0.0114	0.0186	0.0924

### 3.2 Experiment results with retinal prosthesis 1,600-pixel stimulator chip

**Fig 5:**
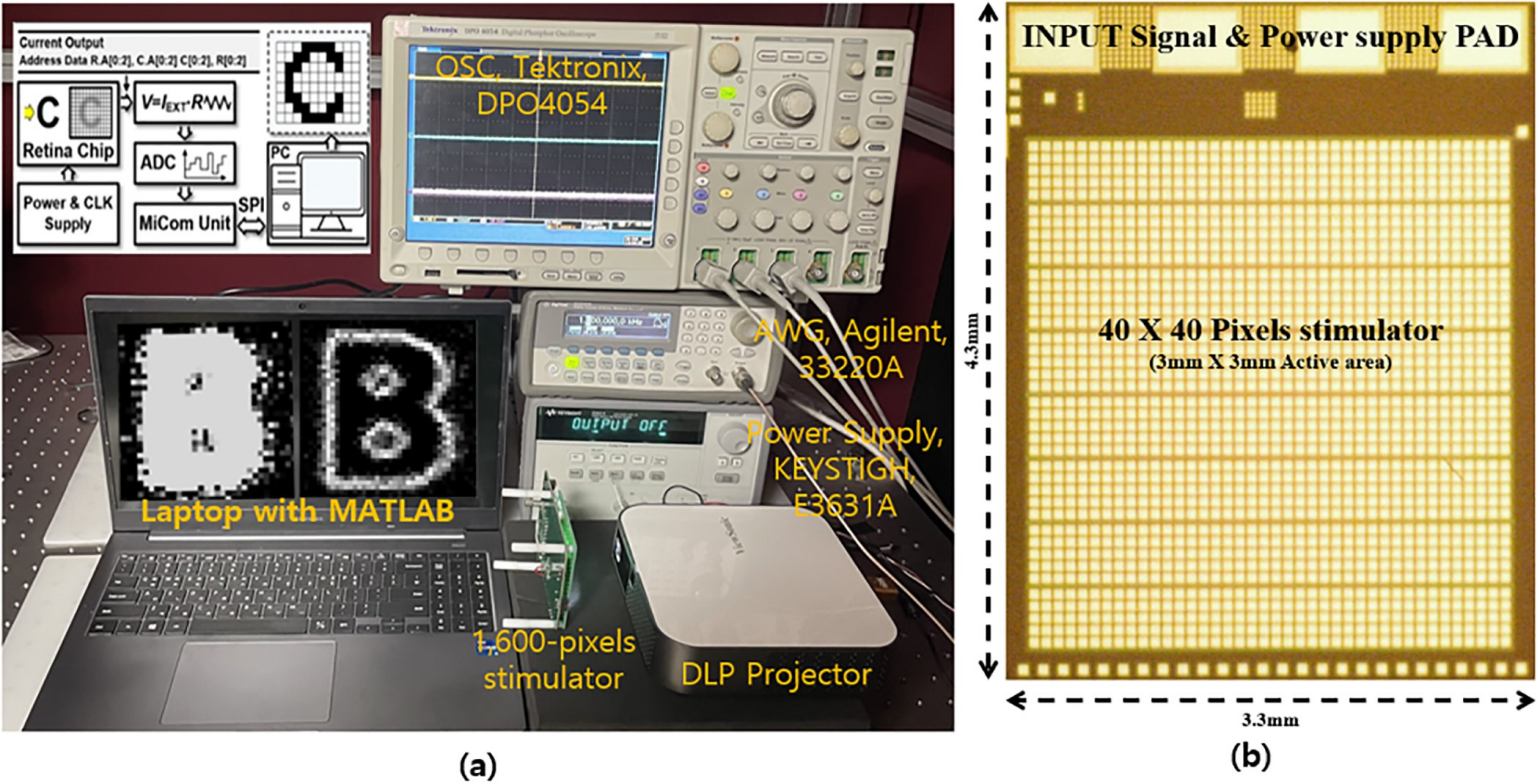
(a) Test bench for measuring the results of images with or without RPED algorithms (b) A microscopic image of the 1,600 pixels stimulator.

Fig 5(a) shows a test bench for measuring the image results based on whether edge detection was applied. The images present a comparison between the traditional light intensity stimulation method and the edge-detection stimulation using the proposed RPED algorithm. Fig 5(b) shows a microscopic image of the 1,600 pixels stimulator that was used in the experiment. This chip comprises individual pixels measuring 80 μm by 84 μm, spaced apart by intervals of 10 μm. This results in a total size of 3.3 mm by 4.3mm. In the experiment, the DLP projector injected a light pattern onto the retinal prosthesis 1600-pixel stimulator chip developed in our previous study [26]. The stimulation current was converted into voltage by adding a resistive feedback trans-impedance amplifier (RTIA: TLC27M, Texas Instruments) outside the chip to conform to the stimulation current generated by the chip owing to image pattern injection. Using an analog-to-digital converter (ADC 104S series, Texas Instruments) that communicates with the microcontrollers, the converted voltage was digitized, and the data were transmitted to the PC. A comparison of the image results between the traditional light intensity stimulation that generates the current dispersion and the RPED algorithm stimulation with improved visual acuity is presented on a laptop screen. Experiments were conducted on a dark optical table to increase the accuracy of the image pattern projection.

**Fig 6:**
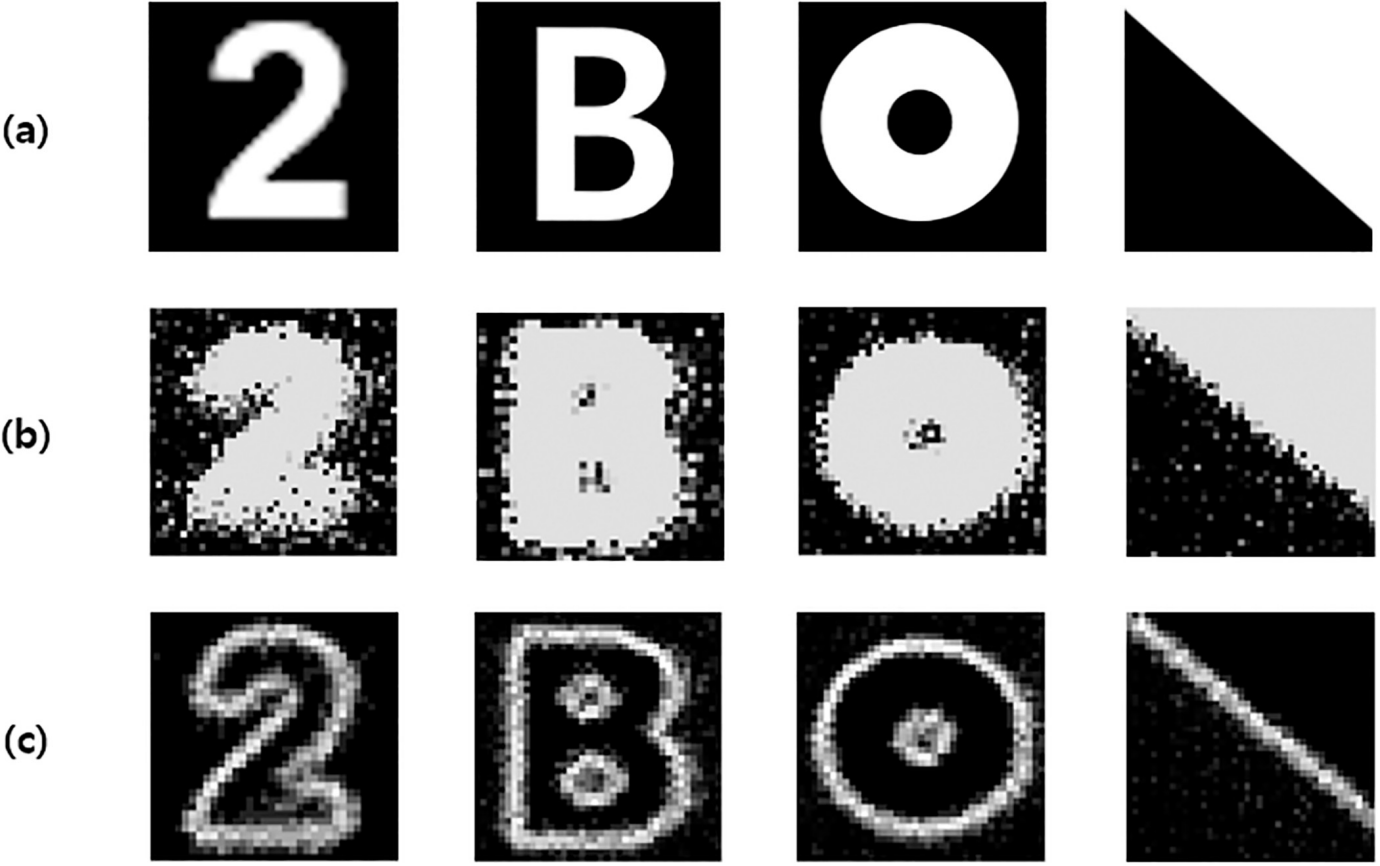
Image results of (a) original image, (b) traditional light intensity stimulation and (c) edge detection stimulation applying RPED algorithm using the retinal prosthesis 1,600-pixel stimulation chip.

Fig 6 shows the MATLAB results of projecting images with different patterns using the laptop, as shown in Fig 5. Fig 6(a) represents the original image used in this experiment. When traditional light intensity stimulation was utilized, all pixels irradiated with light were activated, and ambient noise was generated, as depicted in Fig 6(b). The combination of noise and patterns causes current dispersion, resulting in low spatial resolution owing to the image blur phenomenon. In particular, the image blur phenomenon became more severe in the circular hole-shaped pattern. In addition, as the number of activated pixels increases, power consumption increases proportionally, which can damage normal retinal tissues owing to the heat of the chip. To overcome these problems, the RPED algorithm stimulation was applied with the same values as those of the *α* and Thr mentioned in the result, as shown in Fig 6(c). The RPED algorithm enables cleaner edge detection in simpler images, and better results can be achieved with larger differences in brightness, as illustrated in Fig 6(c). Unlike light intensity stimulation, the results show little noise and clear image patterns can be distinguished, even in the shape of a circular hole. In other words, the spatial resolution is increased, and thermal damage safety is improved by reducing the number of activated pixels. However, edge detection generally requires an edge judgment process that requires more image processing time than traditional light intensity. To reduce the image processing time and power consumption, we propose an RPED algorithm optimized for retinal prostheses, which has better visual acuity and low power consumption. The RPED algorithm has proven to be suitable for retinal prosthesis systems using a 1,600-pixel stimulator chip.

## Conclusion

We proposed and verified the retinal prosthesis edge detection (RPED) algorithm which has enhanced visual acuity and low power consumption, using the MATLAB simulator and 1,600-pixel retinal stimulation chip previously fabricated in DB HiTek 0.18 μm complementary metal-oxide-semiconductor (CMOS) process. To objectively compare the proposed RPED algorithm with other traditional algorithms, such as the conventional edge detection method, Canny, and Sobel, various evaluation indicators, namely MSE, PSNR, and SSIM, were applied. From the experimental results, the proposed RPED algorithm has a higher PSNR of 8.8613 by 1.2751 and a higher SSIM of 0.3441 by 0.1407 compared to the conventional edge detection method. And the processing power consumption during edge detection of the RPED algorithm is 37.5 mW, which is less than the conventional edge detection method by 25 mW. Moreover, the PSNR and SSIM results show that the proposed RPED algorithm is more suitable for a retinal implant application, which requires thousands of simulation pixels, than the Canny and Sobel algorithms, which are generally used in camera image sensors with thousands of megapixels. By virtue of the RPED, which minimizes active pixels, we drastically reduced the active power consumption of the 1,600-pixel stimulator chip that matches the refresh time through multi-pixel activation during stimulations. This demonstration enables both high spatial resolution and low power consumption, which can diminish the secondary heating effect in the retinal tissues. The retinal prosthesis should achieve higher resolution images than what is currently available. However, since the RPED algorithm has not yet been applied to high resolution devices, its effectiveness needs to be evaluated. Additionally, the RPED algorithm maintains the same threshold value even as external images change. Therefore, in future work, we will embed the proposed RPED algorithm that enables automatic threshold adjustment through the introduction of a feedback system via wireless power transmission into a 2000-pixel retinal system-on-a-chip under development, and its performance will be demonstrated in an ex vivo experiment.

## References

[1] Humayun M.S.; Prince M.; De Juan E.; Barron Y.; Moskowitz M.; Klock I.B.; et al.“Morphometric analysis of the extramacular retina from postmortem eyes with retinitis pigmentosa”, *Investig*. *Ophthalmol*. *Vis*. *Sci*, pp. 143–148, 1999. 9888437

[2] Humayun MS, Dorn JD, da Cruz L, Dagnelie G, Sahel JA, Stanga PE, et al “Interim results from the international trial of Second Sight’s visual prosthesis”, *Ophthalmology*, vol. 119, no.4, Apr, 2012. doi: 10.1016/j.ophtha.2011.09.028 22244176 PMC3319859

[3] Yue L, Weiland JD, Roska B, Humayun MS, “Retinal stimulation strategies to restore vision: Fundamentals and systems”, *Prog Retin Eye Res*, vol.53, pp. 21–47, Jul, 2016. doi: 10.1016/j.preteyeres.2016.05.002 27238218

[4] Finn K. E., Zander H. J., Graham R. D., Lempka S. F. and Weiland J. D., "A Patient-Specific Computational Framework for the Argus II Implant," in *IEEE Open Journal of Engineering in Medicine and Biology*, vol. 1, pp. 190–196, 2020. doi: 10.1109/ojemb.2020.3001563 33748766 PMC7971167

[5] Daschner Renate, et al. "Functionality and performance of the subretinal implant chip Alpha AMS." *Sens*. *Mater*. vol. 30, no. 2, pp. 179–192, 2018.

[6] Eberhart Zrenner, Bartz-Schmidt Karl Ulrich Benav Heval, Dorothea Besch, Anna Bruckmann, Gabel Veit-Peter Gekeler Florian, Udo Greppmaier, Alex Harscher, Steffen Kibbel, Johannes Koch, Akos Kusnyerik, Tobias Peters, Katarina Stingl, Helmut Sachs, Alfred Stett, Peter Szurman, Barbara Wilhelm and Robert Wilke, “Subretinal electronic chips allow blind patients to read letters and combine them to words” *Proc*. *R*. *Soc*. *B*, 2011. 10.1098/rspb.2010.1747 PMC3081743 21047851

[7] Stingl K, Bartz-Schmidt KU, Besch D, Braun A, Bruckmann A, Gekeler F, et al“Artificial vision with wirelessly powered subretinal electronic implant alpha-IMS”, *Proc Biol Sci*, Feb, 2013. doi: 10.1098/rspb.2013.0077 23427175 PMC3619489

[8] Cruz L., Dorn J. D., Humayun M. S., Dagnelie G., Handa J., Barale PO., et al“Five-Year Safety and Performance Results from the Argus II Retinal Prosthesis System Clinical Trial”, *Ophthalmology*, Vol. 123, no. 10, pp. 2248–2254, 2016. doi: 10.1016/j.ophtha.2016.06.049 27453256 PMC5035591

[9] Klauke Susanne; Goertz Michael; Rein Stefan; Hoehl Dirk; Thomas Uwe; Eckhorn Reinhard; Bremmer Frank; Wachtler Thomas, “Stimulation with a Wireless Intraocular Epiretinal Implant Elicits Visual Percepts in Blind Humans”, *Investigative Ophthalmology & Visual Science*, vol.52, pp.449–455, Jan, 2011. doi: 10.1167/iovs.09-4410 20861492

[10] Rothermel A. et al., "A CMOS Chip With Active Pixel Array and Specific Test Features for Subretinal Implantation," in *IEEE Journal of Solid-State Circuits*, vol. 44, no. 1, pp. 290–300, Jan, 2009.

[11] Chow AY, Bittner AK, Pardue MT, “The artificial silicon retina in retinitis pigmentosa patients (an American Ophthalmological Association thesis)”, *Trans Am Ophthalmol Soc*, pp. 120–154, Dec, 2010. PMC3016083 21212852

[12] Park Sung Cheol, Park Min Kyu and Kang Moon Gi, "Super-resolution image reconstruction: a technical overview," in *IEEE Signal Processing Magazine*, vol. 20, no. 3, pp. 21–36, May 2003.

[13] Kang H, Abbasi WH, Kim S-W, Kim J., “Fully Integrated Light-Sensing Stimulator Design for Subretinal Implants”, Sensors, vol. 19, 2019 doi: 10.3390/s19030536 30696016 PMC6387200

[14] Opie Nicholas L.; Greferath Ursula; Vessey Kirstan A.; Burkitt Anthony N.; Hamish Meffin; Grayden David B.; et al. “Retinal Prosthesis Safety: Alterations in Microglia Morphology due to Thermal Damage and Retinal Implant Contact”, *Investigative Ophthalmology & Visual Science*, vol. 53, pp. 7802–7812, Nov, 2012. doi: 10.1167/iovs.12-10600 23111605

[15] Opie NL, Burkitt AN, Meffin H, Grayden DB. Thermal heating of a retinal prosthesis: thermal model and in-vitro study. *Annu Int Conf IEEE Eng Med Biol Soc*. 2010. doi: 10.1109/IEMBS.2010.5626670 21096129

[16] Zhou Y.; Liu C.; Huang Y. “Wireless Power Transfer for Implanted Medical Application: A Review”, *Energies*, 2020.

[17] Khalili Moghadam G, Wilke R, Suaning GJ, Lovell NH, Dokos S (2013) “Quasi-Monopolar Stimulation: A Novel Electrode Design Configuration for Performance Optimization of a Retinal Neuroprosthesis:. *PLOS ONE* 8 doi: 10.1371/journal.pone.0073130 23991175 PMC3753255

[18] Wilke RG, Moghadam GK, Lovell NH, Suaning GJ, Dokos S., “Electric crosstalk impairs spatial resolution of multi-electrode arrays in retinal implant”, *J Neural Eng*, 2011. 10.1088/1741-2560/8/4/046016 21673395

[19] Russell, T.L, “Neuronal Circuitry of the Local Edge Detector Retinal Ganglion Cell.” *UC Berkeley*, 2010.

[20] Liu Wentai, Fink W., Tarbell M. and Sivaprakasam M., "Image processing and interface for retinal visual prostheses," *2005 IEEE International Symposium on Circuits and Systems (ISCAS)*, Vol. 3, pp. 2927–2930, 2005.

[21] J. Liu et al., "Saliency-based image processing for retinal prosthesis," *2019 International Conference on Intelligent Informatics and Biomedical Sciences (ICIIBMS)*, pp. 297–297, 2019.

[22] Jeong Hoan Park et al., “1225-Channel Neuromorphic Retinal-Prothesis Soc with Localized Temperature-Regulation”, *2020 IEEE International Solid-State Circuits Conference (ISSCC)*, 2020. 10.1109/TBCAS.2020.3036091 33156793

[23] Eckhorn R., Wilms M., Schanze T., Eger M., Hesse L., Eysel U. T., et al.“Visual resolution with retinal implants estimated from recordings in cat visual cortex,” *Vision Res*., vol. 46, pp. 2675–2690, 2006. doi: 10.1016/j.visres.2006.01.034 16571357

[24] Asher A, Segal WA, Baccus SA, Yaroslavsky LP, Palanker DV., "Image processing for a high-resolution optoelectronic retinal prosthesis", *IEEE Trans Biomed Eng*, 2007. 10.1109/TBME.2007.894828 17554819

[25] Zhou Y.; Liu C.; Huang Y., “Wireless Power Transfer for Implanted Medical Application: A Review”, *Energies*, vol. 13, no. 11, 2020.

[26] Fotopoulou K. and Flynn B. W., "Wireless Power Transfer in Loosely Coupled Links: Coil Misalignment Model," in *IEEE Transactions on Magnetics*, vol. 47, no. 2, pp. 416–430, Feb. 2011.

[27] Agarwal K., Jegadeesan R., Guo Y. -X. and Thakor N. V., "Wireless Power Transfer Strategies for Implantable Bioelectronics," in *IEEE Reviews in Biomedical Engineering*, vol. 10, pp. 136–161, 2017. doi: 10.1109/RBME.2017.2683520 28328511

[28] Kang H.; Kim J.; Kim J, “Integrated High-Temporal-Resolution and High-Density Subretinal Prosthesis Using a Correlated Double-Sampling Technique”, *Sensors*, vol. 23, no. 14, 2023. doi: 10.3390/s23146501 37514794 PMC10383336

[29] Chenais N.A.L., Airaghi Leccardi M.J.I. & Ghezzi D. Photovoltaic retinal prosthesis restores high-resolution responses to single-pixel stimulation in blind retinas. *Commun Mater* 2, 28 (2021).

[30] Image I.J., “Edge Detection Operators: Peak Signal to Noise Ratio Based Comparison”, International Journal of Image, Graphics and Signal Processing, vol. 6, no. 10, pp. 55–61, 2014.

[31] A. Horé and D. Ziou, "Image Quality Metrics: PSNR vs. SSIM," *2010 20th International Conference on Pattern Recognition*, pp. 2366–2369, 2010.

[32] Vincent Olufunke Rebecca and Folorunso Olusegun. “A Descriptive Algorithm for Sobel Image Edge Detection.”, *InSITE 2009*, vol. 9, 2009.

[33] Canny J., "A Computational Approach to Edge Detection," in *IEEE Transactions on Pattern Analysis and Machine Intelligence*, vol. PAMI-8, no. 6, pp. 679–698, Nov. 1986. 21869365

[34] R. C. Gonzalez and R. E. Woods, “Digital Image Processing”, *PrenticeHall*, 2002.

[35] X. Zhu, M. Tang, K. Zhang and Q. Wang, "Image detection method based on improved Canny algorithm," *2021 40th Chinese Control Conference (CCC)*, 2021.

[36] Deriche R., “Using Canny’s criteria to derive a recursively implemented optimal edge detector”, *Int*. *J*. *Computer Vision*, vol. 1, pp. 167–187, 1987.

